# The survivor strain: isolation and characterization of *Phormidium yuhuli* AB48, a filamentous phototactic cyanobacterium with biotechnological potential

**DOI:** 10.3389/fbioe.2022.932695

**Published:** 2022-08-15

**Authors:** Moritz Koch, Avery J. C. Noonan, Yilin Qiu, Kalen Dofher, Brandon Kieft, Soheyl Mottahedeh, Manisha Shastri, Steven J. Hallam

**Affiliations:** ^1^ Department of Microbiology and Immunology, University of British Columbia, Vancouver, BC, Canada; ^2^ Genome Science and Technology Program, University of British Columbia, Vancouver, BC, Canada; ^3^ ECOSCOPE Training Program, University of British Columbia, Vancouver, BC, Canada; ^4^ Graduate Program in Bioinformatics, University of British Columbia, Vancouver, BC, Canada; ^5^ AlgaBloom International Ltd., Richmond, BC, Canada; ^6^ Life Sciences Institute, University of British Columbia, Vancouver, BC, Canada

**Keywords:** cyanobacteria, phormidium, photobioreactor, isolate, stress tolerance, oscillatoriales

## Abstract

Despite their recognized potential, current applications of cyanobacteria as microbial cell factories remain in early stages of development. This is partly due to the fact that engineered strains are often difficult to grow at scale. This technical challenge contrasts with the dense and highly productive cyanobacteria populations thriving in many natural environments. It has been proposed that the selection of strains pre-adapted for growth in industrial photobioreactors could enable more productive cultivation outcomes. Here, we described the initial morphological, physiological, and genomic characterization of *Phormidium yuhuli* AB48 isolated from an industrial photobioreactor environment. *P. yuhuli* AB48 is a filamentous phototactic cyanobacterium with a growth rate comparable to *Synechocystis* sp*.* PCC 6803. The isolate forms dense biofilms under high salinity and alkaline conditions and manifests a similar nutrient profile to *Arthrospira platensis* (*Spirulina*). We sequenced, assembled, and analyzed the *P. yuhuli* AB48 genome, the first closed circular isolate reference genome for a member of the *Phormidium* genus. We then used cultivation experiments in combination with proteomics and metabolomics to investigate growth characteristics and phenotypes related to industrial scale cultivation, including nitrogen and carbon utilization, salinity, and pH acclimation, as well as antibiotic resistance. These analyses provide insight into the biological mechanisms behind the desirable growth properties manifested by *P. yuhuli* AB48 and position it as a promising microbial cell factory for industrial-scale bioproduction[**221, 1631**].

## Introduction

For several decades, there have been high hopes for the application of blue-green algae (cyanobacteria) as bioproduction platforms (Branco dos Santos et al., 2014). Their innate capacity to fix carbon dioxide (CO_2_) into biomass, while harnessing light energy provided by the Sun, makes them promising microbial cell factories for sustainable bioproduction of energy and materials (Hudson et al., 2022). Examples of cyanobacterial engineering for increased biosynthesis of commodity compounds include the production of biofuels (Nozzi et al., 2013), bioplastics (Koch et al., 2019; Koch et al., 2020), and high-value products, such as squalene (Dienst et al., 2020). However, most of these examples have relied on model chassis *Synechocystis sp*. PCC 6803 and *Synechococcus* sp. (Lai and Lan, 2015). Despite extensively developed genetic systems and a detailed understanding of their metabolic properties, these strains have proven difficult to cultivate at scale (Panuschka et al., 2019; Price et al., 2022).

Interestingly, this technical challenge contrasts with the dense and highly productive cyanobacteria populations thriving in many natural environments. It has been proposed that the selection of strains pre-adapted for growth in industrial photobioreactors could enable more productive, and therefore more economical, cultivation outcomes. Non-model filamentous cyanobacteria, including *Arthrospira platensis* (*Spirulina*), are becoming increasingly important for industrial bioproduction and biological engineering applications (Taton et al., 2020). This is a result of their capacity to form dense biofilms and, in some cases, their natural resilience to contamination due to unimpeded growth under high salinity and alkalinity conditions (Nies et al., 2017; Ataeian et al., 2021). In addition to *A. platensis*, distantly related lineages of fast-growing filamentous cyanobacteria including *Phormidium* sp*.*, show biotechnological potential. *Phormidium* are common denizens of soda lakes and other high-salinity environments, forming dense biofilms in conditions up to 200 g/L total salinity and pH 11.2 (Kupriyanova et al., 2016; Samylina et al., 2014; Ataeian et al., 2021). Additional beneficial traits include the capacity to fix atmospheric nitrogen, store phosphate, and produce a significant quantity of extracellular polysaccharides (EPSs) (Nies et al., 2017; Ataeian et al., 2021). *Phormidium* species show stable growth in outdoor, non-sterile photobioreactor environments, and are considered promising candidates for industrial bioproduction (Haines et al., 2022). They have also been used as a phytostimulant in wheat cultivation, demonstrating potential use as fertilizer supplements (Hussain and Hasnain, 2011).

Here, we described the initial morphological, physiological, and multi-omic (DNA, protein, and metabolites) characterization of *Phormidium yuhuli* AB48, isolated from an industrial photobioreactor environment initially designed to grow *Spirulina*. We first described the isolation of *P. yuhuli* AB48 from its original biofilm community, followed by microscopy and stress tests to constrain limits on growth and to characterize mechanisms behind growth phenotypes relevant to biotechnological applications. Nutritional and compositional profiling is complemented by a more focused look at the gene content and expression related to observed growth phenotypes.

## Materials and methods

### Chemicals and growth media

All chemicals were purchased from Sigma-Aldrich (Germany) unless indicated otherwise. For the preparation of the Zarrouk medium (ZM), the following ingredients are added: 1 L ddH_2_O: 16.8 g NaHCO_3_, 1 g NaCl, 0.2 g MgSO_4_.7H_2_O, 0.01 g FeSO_4_.7H_2_O, 1 g K_2_SO_4_, 0.04 g CaCl_2_.2H_2_O, 2.5 g NaNO_3_, and 0.5 g K_2_HPO_4_, to 1 ml Hutners’s Solution (Chlamydomonas Resource Center, United States). Once all ingredients were combined, they were mixed until all chemicals were in solution. Subsequently, the solution was filter sterilized. Alternatively, 100x stock solution of the individual ingredients were prepared, autoclaved, and combined under sterile conditions.

### Cultivation of cyanobacterial strains

For standard cultivation, isolated *P. yuhuli* AB48 was incubated in 100 ml Erlenmeyer flasks filled with 50 ml ZM. For growth on solid surfaces, agar plates were prepared by adding 1.5% agar to the respective medium. Cyanobacteria were cultivated under continuous light at 30°C, unless stated otherwise. As a light source, Metalux Flushmount lamps equipped with LEDs were used. The light temperature was set to 4000 k and the intensity was ∼50 photons m^−2^ s^−1^. The cultures were shaken continuously at 120 rpm, unless otherwise stated. Antibiotic working concentrations for relevant experiments are: streptomycin (50 μg/ml), apramycin (50 μg/ml), chloramphenicol (25 μg/ml), tetracycline (10 μg/ml), kanamycin (50 μg/ml), ampicillin (100 μg/ml), carbenicillin (100 μg/ml), and gentamicin (20 μg/ml).

### DNA extraction and purification

DNA was extracted from biofilms and laboratory cultures using a CTAB-chloroform extraction protocol adapted from published protocols (William et al., 2004; Morin et al., 2010). The culture volume was scaled from 1 to 25 ml in 1.5 ml or 50 ml tubes, depending on the application and desired DNA yield. Volumes indicated in this protocol represent those used in 1.5 ml microcentrifuge tubes and 50 ml tubes, respectively [*1.5 ml: 50 ml*]. The cells were pelleted at 4000 × *g* for 10 min and resuspended in a resuspension buffer (0.15 M NaCl and 0.1 M EDTA pH 8.1) [*1 ml: 20 ml*]. This was repeated two times to wash cells. After the final wash, the cells were again resuspended in resuspension buffer [*0.5 ml: 10 ml*], before two freeze–thaw cycles, alternating between liquid N_2_ and a 37°C water bath. Thawed cells should not be stored at 37°C longer than necessary for thawing. Subsequently, 100 mg/ml lysozyme (Sigma-Aldrich) [*0.125 ml: 1.25 ml*] and 20 mg/ml RNase A (NEB) [*2 μL: 20 μL*] were added to the sample, before incubation rotating for 30 min at 20 °C. 600 mAU/mL Proteinase K (Millipore) [*40 μL: 400 μL*] and 20% SDS [*150 μL: 1.5 ml*] were added to the lysate before incubation rotating at 55 °C for 1 h. Lysates in 1.5 ml tubes were split into 2 × 550 μL for subsequent steps. Then, 5 M NaCl [*150 μL: 4.5 ml*] and 10% CTAB w/v [*70 μL: 2.5 ml*] were added to the lysate and incubated at 65°C for 10 min. DNA isolation was performed by adding one volume of 25:1 chloroform:IAA (isoamyl alcohol) to lysates and mixing by gentle inversion. Prior to this step, lysates may need to be split into additional tubes if the capacity of tubes is insufficient. The mixtures were incubated on the ice for 30 min before centrifuging at 6000 × *g* for 10 min (13,000 × *g* was used with 1.5 ml microcentrifuge tubes). The aqueous fraction was removed after centrifugation, followed by ethanol precipitation. The pellets were washed three times with 70% ethanol and resuspended in a Tris-EDTA buffer. Quality and yield were determined using a NanoDrop spectrophotomer and the Quant-iT PicoGreen dsDNA Assay (Invitrogen). This extraction protocol was used for all DNA extractions, unless otherwise noted.

### Community structure analysis

The community structure was determined using amplicon sequencing. DNA was extracted from relevant samples using the protocol described earlier. Dual-indexing, one-step 10 µL PCR reaction is performed on a LabCyte Access Workstation using Quanta 5PRIME HotMasterMix with 1 ng input DNA and complete “fusion primers,” that include Illumina Nextera adapters and indices and specific regions targeting the V4/V5 region of the small subunit ribosomal RNA (SSU or 16S rRNA) gene (Comeau et al., 2017). Amplicons are quantified using a picogreen assay (Quant-iT™ PicoGreen™ dsDNA Assay Kit, ThermoFisher) and 2 ng of each product was pooled for subsequent cleanup using the AmpureXP PCR cleanup protocol (Beckman). The pooled library was quantified using a picogreen assay and loaded onto an Illumina MiSeq Reagent Kit v3 (600-cycle) using the manufacturer’s recommendations with 10% PhiX. Data processing and taxonomic classification were performed using Qiime2 (v2020.11.1), and read quality control was performed using the dada2 (v2020.11.1) plugin. Taxonomic classifications were performed using the gg-13-8-99-515-806-nb-classifier Naive Bayes classifier, trained on the Greengenes 16S rRNA gene database. Figures were prepared in R (v4.0.3) using the tidyverse (v1.3.1) and ggplot2 (v3.3.5) packages.

### Isolation of axenic *P. yuhuli* AB48

To isolate the strain, a protocol was developed based on the ability of *Phormidium* sp. AB48 to survive under high salinity and alkalinity conditions. A biomass sample was obtained from AlgaBloom and cultivated in ZM for several weeks prior to dispensing into a 48-well gradient plate containing increasing concentrations of sodium chloride (NaCl) and sodium hydroxide (NaOH) (Supplementary Figure S1). Rows contained between 0 and 150 mM of NaOH, while columns contained between 0 and 672 mM of NaCl. After two days of incubation under Metalux Flushmount lamps as described earlier, several wells which showed only a faint green color (indicating limited cyanobacterial growth) were selected. The content of these wells was transferred into fresh ZM, passed several times, and then checked *via* microscopy and amplicon sequencing until an isolate subsequently named *P. yuhuli* AB48 was confirmed.

### Isolate growth tests

An artificial urine medium (AU) was used to test growth on an alternative nitrogen source (Sarigul et al., 2019). For this, nitrogen-free ZM (without NaNO_3_) was prepared and combined with AU in different concentrations. When testing sensitivity to various antibiotics, 100 μL of axenic and homogenized *P. yuhuli* AB48 was added to each well of a 96-well microplate excluding the outermost wells (which were filled with distilled water). Then, 100 μL of antibiotic/ZM dilution was combined with the culture in a randomized order (to account for lighting/position associated growth effects) and mixed by reverse pipetting. The first group of antibiotics consisted of tetracycline, ampicillin, and apramycin and a second trial included chloramphenicol, streptomycin, and tetracycline (as the initial stock appeared slightly discolored). Optical density (OD) readings of the microplates were taken on a PHERAstar FSX (BMG) plate reader using the spiral average measurement as the rapid clumping of *P. yuhuli* AB48 makes single OD readings inaccurate. Plates were, then, incubated under conditions described earlier and additional OD measurements were taken approximately every 24 h for 5 days.

### Sample preparation and growth measurements

To measure the culture cell density, the suspension was first homogenized for 40 s using a Vevor Fsh-2a homogenizer. From this, 100 µL were taken and diluted 1:10 with ZM. Subsequently, the optical density was measured at 750 nm using a Thermo Scientific Evolution 60S photospectrometer.

### Microscopy and cell imaging

The morphology of *P. yuhuli* AB48 was described using light and scanning electron microscopy (SEM) techniques. For brightfield images, a Zeiss Axio Observer 7 microscope with an attached Calibiri 7 light source and an Axiocam 702 mono camera was used. For the detection of autofluorescence, a mRF12 filter was used. The samples imaged using SEM were isolated from suspension and filtered onto 0.2 µm Nucleopore filters where they were fixed with 4% formaldehyde/2% glutaraldeyde in 0.05 M, pH 7.4 sodium cacodylate. The cells were washed 3x with fixative-free buffer, then post-fixed with 1% buffered OsO_4_ before multiple washes with distilled, deionized water. Following the washes, the samples were stacked in a stainless-steel filter holder, and taken through a staged, microwave dehydration using 1 min at power-level 3 (∼180W) in 30, 50, and 70% ethanol. The samples were kept at 70% ethanol overnight at −20°C. Following overnight dehydration, the samples were treated for 1 min PL3 at 80, 90, and 95% ethanol and finally for 3 min PL3 at 100% dry ethanol for dehydration, twice. The final dehydration step was performed for 10 min at room temperature in 100% dry ethanol. The samples were then critically point-dried (Tousimis Auto-Sam Dri 815B) mounted to aluminum SEM stubs using double-sided tabs, sputter coated with 10 nm gold (Cressington 208HR), and imaged on a Hitachi S2600 VP-SEM.

### Chemical analysis

To analyze the chemical composition of *P. yuhuli* AB48, several liters of culture were harvested *via* centrifugation (4000 × *g*, 15 min). The pellet was washed twice with distilled water to remove residual components of the growth medium. The condensed biomass was, then, transferred on a metal tray and incubated in an oven at 60°C for several hours until the sample was completely dry. The dried biomass was then shipped to MB Labs (Sydney, Canada), where elemental composition and potential toxin production were determined.

### Genomic analysis

Genomic DNA was extracted using the CTAB-chloroform extraction protocol as described earlier and sequenced on Illumina HiSeq and Oxford Nanopore MinION Mk1B platforms. In order to account for over-sequencing of the sample and improved assembly accuracy, we subsampled 283,172,660 Illumina paired-end sequencing reads (42475.9 Mbp) using BBMap (v38.18), with sample seed set as 3265. This resulted in a subsample of 5,659,080 reads (848.9 Mbp). These reads were trimmed by BBDuk (v38.93), with default settings, removing 2818 reads (4.3 Mbp). Base calling of 130,887 nanopore reads (834.3 Mbp) was performed using Guppy (v5.0.16) with the high-accuracy model and default settings. A total of 64,617 reads (410.8 Mbp) passed the quality filtering (Q > 10). Adapters were trimmed using Porechop (v0.3.2) with default settings. The combined sort- and long-read data were hybrid assembled using the Unicycler with default parameters (Wick et al., 2017). Two circular contigs were resolved corresponding to the isolated cyanobacterial genome and associated plasmid. Open reading frames (ORFs) and non-coding features (e.g., tRNA and rRNA) were predicted using PROKKA (v1.14.5) (Seemann, 2014). In addition to the UniProt database annotations provided by PROKKA (Consortium, 2020), predicted ORFs were also annotated using *hmmsearch* in HMMER (v3.3.2) queried against the Pfam database (v35.0) (Mistry et al., 2020) and RPS-BLAST (v2.12.0) (Yang et al., 2020) queried against the Cluster of Orthologous Genes (COG) (Galperin et al., 2020) database (downloaded Jan. 16, 2022). Resulting annotations were used to identify peptides in the proteomic analysis (described below) and for gene cluster analysis. Synteny plots were prepared in R (v4.0.3) using the gggenes (v0.4.1) package. Ribosomal rRNA genes were extracted from the genome using the program barrnap (Seemann, 2022).

### Phylogenetic analysis

A concatenated marker gene phylogeny approach was employed to understand evolutionary relationships between described cyanobacterial genomes and *Phormidium yuhuli* AB48. A set of 12 high-quality isolate genomes were selected from representative cyanobacteria species in the Genome Taxonomy Database (GTDB) (Parks et al., 2018), along with the close 16S rRNA relative candidate species *Phormidium alkaliphilum* (Supplementary Table S1). The software program cognac (Crawford and Snitkin, 2021) was used to select a set of single-copy marker genes shared between these genomes using a core gene threshold of 90% (all but one genome must contain the protein ortholog) and an amino acid percent identity and alignment coverage threshold of at least 70% for each orthologous group. The resulting set of amino acid sequences was concatenated, aligned with muscle (Edgar, 2004), and trimmed using ClipKIT (Steenwyk et al., 2020). A maximum-likelihood phylogenetic tree with bootstrap values was built from the trimmed alignment using FastTree (Price et al., 2010). Based on this phylogeny, *P. yuhuli* AB48 belongs to the GTDB *Phormidium*_A genus. Therefore, the process of concatenated marker gene tree building was repeated using only the GTDB genomes in this taxon to determine finer-scale phylogenetic resolution (with cognac thresholds set to 90% for all three parameters). Furthermore, the pairwise average nucleotide identity (ANI) between *P. yuhuli* AB48 and the members of this genus was calculated using FastANI (Jain et al., 2018).

### Proteomic and metabolomic sample preparation

The cell pellets were frozen in liquid N_2_ and stored at -80 °C before processing. The samples were then lyophilized after being frozen. Following lyophilization of the pellets, 3 mg of sample was weighed into 1.7 ml microcentrifuge tubes (Sorenson bioscience, Salt Lake City, UT). To lyse samples, 270 µL cold (−20°C) methanol:water mix (prepared 4:3 (v/v)) was added and a spiral pestle grinder was used for 10–15 s. An additional 200 µL of cold 4:3 MeOH:H_2_O was used to rinse the spiral pestle into the sample tube. Then, 430 µL cold (−20°C) chloroform was added to each sample to obtain a ratio of 8:4:3 chloroform:methanol:water and vigorously vortexed for 30 s. The sample was, then, placed back on the ice for 5 min and then vortexed for 30 s followed by centrifugation at 10,000 *x g* for 10 min at 4°C. The entire upper polar phase and 100 µL of the lower non-polar phase were collected into a tared glass vial for metabolomics analysis. The remaining (∼400 µL) non-polar phase was collected into a separate glass vial and stored for future lipidomics analysis. The protein interlayer was washed twice with 500 µL of cold 100% methanol. Then, 250 µL of an 8 M urea in a 100 mM ammonium bicarbonate solution was added to the protein pellet and vortexed to dissolve. Following protein estimation using a bicinchoninic acid (BCA) assay (Thermo Scientific, Waltham, MA United States), dithiothreitol (DTT) was added to obtain 5 mM concentration and incubated at 60°C for 30 min with constant shaking at 850 rpm. The samples were then diluted 8-fold and prepared for digestion with 100 mM NH_4_HCO_3_, 1 mM CaCl_2_ and sequencing grade trypsin (USB, Santa Clara, CA) was added to samples at a 1:50 (w/w) trypsin-to-protein ratio and digested for 3 h at 37°C. SPE using Discovery C18 50 mg/1 ml columns (Supelco, St.Louis, MO) was performed to complete the preparation of samples for MS analysis. The columns were conditioned with 3 ml each of methanol and 0.1% trifluoroacetic acid (TFA) in H_2_O. The samples were then loaded onto each column followed by 4 ml of 95:5: H_2_O:ACN, 0.1% TFA. The samples were eluted with 1 ml 80:20 ACN:H_2_O, 0.1% TFA, and concentrated down to ∼100 µL using a Speed Vac. The peptide concentration was estimated again and samples were diluted to 0.10 μg/μL with nanopure water for proteomics LC–MS/MS analysis.

### Proteomic and metabolomic data generation and analysis

*Proteomics*: MS analysis was performed using a Q-Exactive HF-X mass spectrometer (Thermo Scientific) outfitted with a homemade nano-electrospray ionization interface. Electrospray emitters were homemade using 150 μm o.d. X 20 μm i.d. chemically etched fused silica (Kelly et al., 2006). The ion transfer tube temperature and spray voltage were 320°C and 2.2 kV, respectively. Data were collected for 120 min following a 60 min delay from sample injection. FT-MS spectra were acquired from 300 to 1800 m/z at a resolution of 60 k (AGC target 3e6) and the top 16 FT-HCD-MS/MS spectra were acquired in a data-dependent mode with an isolation window of 0.7 m/z at a resolution of 30 k (AGC target 2e5) using a normalized collision energy of 30 and 45 s exclusion time for charge states from 2 to 6. Generated MS/MS spectra from sample measurements (4 replicates) were searched for peptides using the mass spectral generating function (MSGF+) algorithm (Kim et al., 2008; Kim and Pevzner, 2014) against the *Phormidium yuhuli* AB48 genome, with 16 common contaminant sequences added. MSGF+ was used in a target/decoy mode with 20 ppm parent ion tolerance, partial tryptic rule, and methionine oxidation (+15.9949). Best matches from the MSGF + searches were filtered at 1% FDR and only protein-specific peptides were used in consequent aggregation and quantitative analysis. Relative peptide abundances can be determined by calculating the area under the curve of the peptide ion peak in the MS measurement. This was accomplished using MASIC software (Monroe et al., 2008) and results were aggregated using the MS SQL (Microsoft) database.

*Metabolomics*: The metabolite fraction was dried under a vacuum to remove moisture and was chemically derivatized and analyzed as previously reported (Kim et al., 2015; Pomraning et al., 2021). Briefly, the extracted metabolites were derivatized by methoxyamination and trimethylsilyation (TMS), and then the samples were analyzed by GC–MS. The samples were run in an Agilent GC 7890A using a HP-5MS column (30 m × 0.25 mm x 0.25 μm; Agilent Technologies, Santa Clara, CA) coupled with a single quadrupole MSD 5975C (Agilent Technologies). One microliter of sample was injected into a splitless port at constant temperature of 250°C. The GC temperature gradient started at 60°C, with a hold of temperature for 1 min after injection, followed by an increase to 325°C at a rate of 10°C/min and a 5 min hold at this temperature. A fatty acid methyl ester standard mix (C8-28) (Sigma-Aldrich) was analyzed in parallel as standard for retention time calibration. GC–MS raw data files were processed using the Metabolite Detector software (Hiller et al., 2009). Retention indices (RI) of detected metabolites were calculated based on the analysis of a FAME mixture, followed by their chromatographic alignment across all analyses after deconvolution. Metabolites were initially identified by matching experimental spectra to a PNNL extended version of Agilent GC-MS metabolomics Library, containing spectra and validated retention indices for over 850 metabolites. Then, the unknown peaks were additionally matched using the NIST17/Wiley11 GC–MS library. All metabolite identifications and quantification ions were validated and confirmed to reduce deconvolution errors during automated data-processing and to eliminate false identifications.

Downstream analysis of metabolomic and proteomic data was performed in R (v4.0.3) using the tidyverse (v1.3.1). Figures were prepared using ggplot2 (v3.3.5) and WeightedTreemaps (v0.1.1) packages.

## Results

**FIGURE 1:**
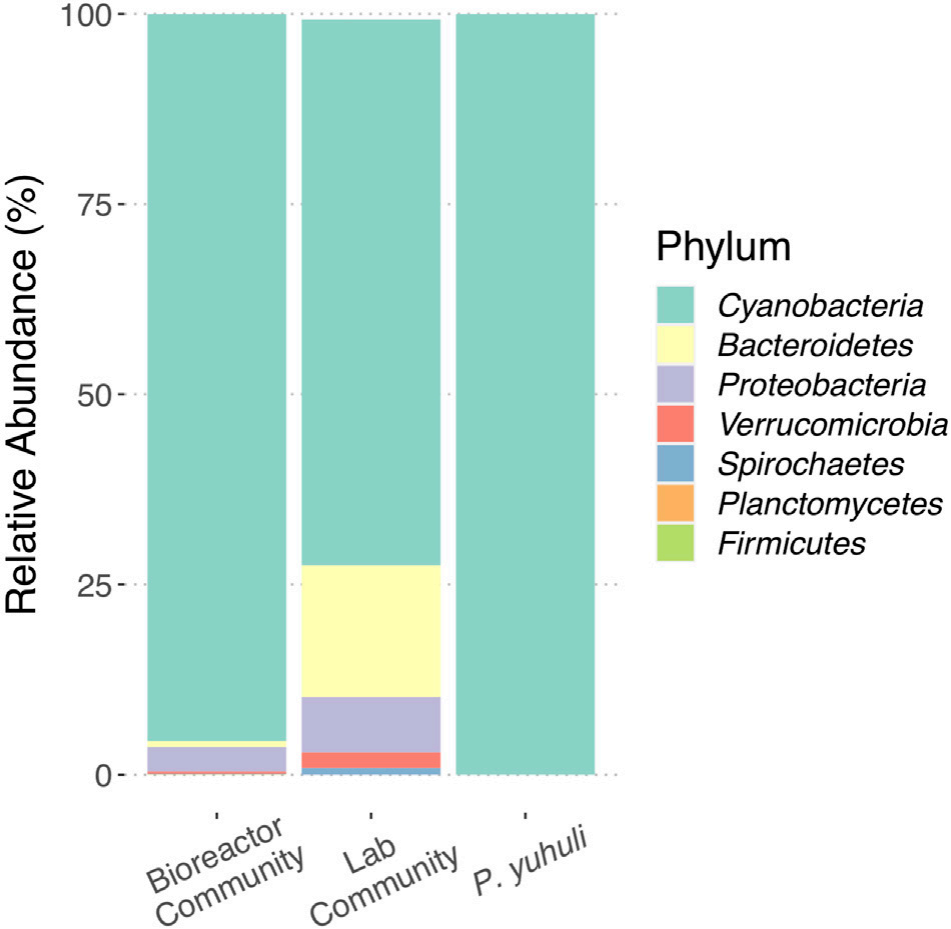
Profiling the photobioreactor community and *Phormidium* sp. AB48 isolate. Comparison of ASV proportions identified in biofilm source, laboratory culture, and axenic culture.

Biofilm biomass was obtained from an industrial photobioreactor operated by AlgaBloom International Ltd., a biotechnology company in British Columbia Canada that uses high salinity and alkaline conditions to support biofilm-based cyanobacterial growth, including *Spirulina*. Using light microscopy, a filamentous cyanobacterium was observed to dominate the biofilm, but its morphology was distinct from *Spirulina*. DNA was extracted from the biofilm and 16S rRNA gene amplicon sequencing was used to investigate the biofilm community structure. In parallel, a laboratory culture of the biofilm was propagated and profiled for comparison. Amplicon sequence variants (ASVs) identified in both source and culture samples indicated that cyanobacteria affiliated with *Phormidium* sp. represented ∼95 and ∼75% of ASVs associated with source and culture samples, respectively (Figure 1). The source sample also contained ASVs affiliated with *Bacteroidetes*, *Proteobacteria*, and *Verrucomicrobiota* in small proportions. The same taxonomic groups were identified in the culture sample, albeit at increased proportions, with additional ASVs affiliated with *Spirochaetes*, *Planctomycetes*, and *Firmicutes* identified (Figure 1).

### Strain isolation

A method was developed to isolate the *Phormidium* sp. strain using a matrix of increasing salinity and alkalinity in 48-well plates (Supplementary Figure S1). The cells growing under high salinity (672 mM NaCl) and pH (100 mM NaOH), which was the harshest condition in which growth was observed, were selected and passaged several times in a fresh ZM medium. Subsequent observations using microscopy and 16S rRNA gene amplicon sequencing indicated an axenic culture of *Phormidium* sp. AB48, subsequently named *Phormidium yuhuli* AB48 (Figure 1).

### Genome sequencing and species identification

Genomic DNA was extracted from the axenic culture using a CTAB-chloroform extraction protocol (William et al., 2004; Morin et al., 2010) and sequenced on Illumina HiSeq and Oxford Nanopore MinION platforms. Sequencing datasets were hybrid assembled, resulting in a 4,747,469 bp closed reference genome (51.68% GC), predicted to encode 4,239 genes, and a 51,599 bp circular plasmid (48.61% GC) (Table 1). The isolated genome and plasmid were 99.99% similar to assembled sequences sourced directly from the original AB48 biomass provided by AlgaBloom (article in preparation). We identified two identical full-length 5S, 16S rRNA, and 23S rRNA gene sequences in the *Phormidium* sp. genome. This duplication is a common feature of cyanobacterial genomes (Schirrmeister et al., 2012) and was also identified in closely related *Phormidium* species. The full-length 16S rRNA gene sequence 1,463 bp in length was queried against the prokaryotic 16S rRNA gene database from the NCBI RefSeq Targeted Loci Project using blastn. When filtered by sequence identity, the best representatives are *Sodalinema komarekii* strain PMC 869.14 (90.0% coverage, 99.3% identity) and ​​*Baaleninema simplex* strain PCC 7105 (96.0% coverage, 95.5% identity).

**TABLE 1:** *P. yuhuli* AB48 genome features in comparison to related reference genomes.

Species	*Phormidium yuhuli*	*Phormidium alkaliphilum*	*Phormidium lacuna* HE10JO	*Synechocystis* sp. PCC 6803
Genome size (Mb)	4.810	5.000	4.819	3.947
GC content (%)	51.63%	51.70%	51.34%	47.37%
Gene count	4239	4409	4164	-
CDS count	4184	4355	4101	3713
CheckM completeness	100.00	99.73	99.73	99.78
CheckM contamination	0.54	0.00	0.00	0.00
Contigs count	2 (including plasmid)	1	104	5
Longest contig	4758454	5000054	145031	3573470
n50	4758454	5000054	82333	3573470
tRNAs	48	47	50	41
5S rRNA	2	2	4	0
16S rRNA	2	2	4	2
23S rRNA	2	2	4	2
tmRNA	1	1	1	-
Accession	CP098611	CP075902.1	GCF_900149785	GCA_000009725.1

**FIGURE 2:**
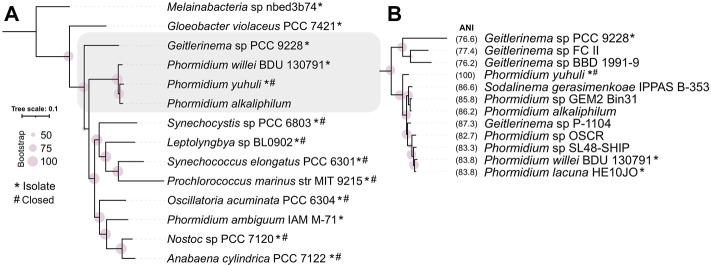
Phylogenetic classification of *Phormidium yuhuli* AB48 using concatenated marker genes and average nucleotide identity (ANI). **(A)** Classification of 12 high-quality isolate genomes of representative cyanobacterial species selected from GTDB, with *P. yuhuli* AB48 and *P. alkaliphilum*, places *P. yuhuli* AB48 an Oscillatoriales in the GTDB *Phormidium_A* genus. **(B)** Classification of members of *Phormidium_A* and closely related *Geitlerinema* genus, and ANI computation, indicate that *Geitlerinema* sp. P-1104 is the closest related species to *P. yuhuli* AB48. Symbols “*” and “#” indicate whether genomes are from isolates and closed, respectively.

To resolve the phylogenetic placement of the isolate genome, a maximum likelihood tree was constructed using a concatenated set of 120 single-copy marker genes shared between *P. yuhuli* AB48 and high-quality isolate reference genomes in GTDB (Figure 2A). This phylogeny suggested that *P. yuhuli* AB48 belongs to the GTDB *Phormidium*_A genus, with the closest relative among selected isolated genomes identified as *Phormidium willei* (ANI 83.8%). All members of this genus, including several isolates (*Phormidium lacuna HE10JO (* *Nies et al., 2017* *)*, *Sodalinema gerasimenkoae* sp. *(* *Kupriyanova et al., 2016* *)*, *Phormidium willei* BDU 130791, and *Geitlerinema* sp. *P-1104*) and several metagenome-assembled genomes (*Phormidium* sp. SL48-SHIP 9 (Rozanov et al., 2019) *Phormidium* sp.OSCR (Nelson William et al., 2016), and *Phormidium alkaliphilum (* *Ataeian et al., 2021* *)*) have draft genomes*.* To further resolve the phylogenetic context of *P. yuhuli* AB48, another maximum likelihood tree was constructed from a more stringently defined set of 50 concatenated, single-copy marker genes shared amongst *P. yuhuli* AB48 and the draft genomes in this genus. This phylogeny indicated an internal but basal relationship to members of this genus, with *P. yuhuli* AB48 ANI values of 86.2% to *Candidatus Phormidium alkaliphilum* and 87.3% to *Geitlerinema* sp. P-1104, its closest apparent relative among sequenced genomes (Figure 2B). Based on this result, *Phormidium* sp. AB48 was designated as a new species with the name *Phormidium yuhuli* AB48. The word yuhuli is derived from hən̓q̓əmin̓əm̓, the language of the Musqueam First Nation, and means one who persists or survives, in reference to the competitive advantage observed in the photobioreactor setting.

### Morphology and growth characteristics

**FIGURE 3:**
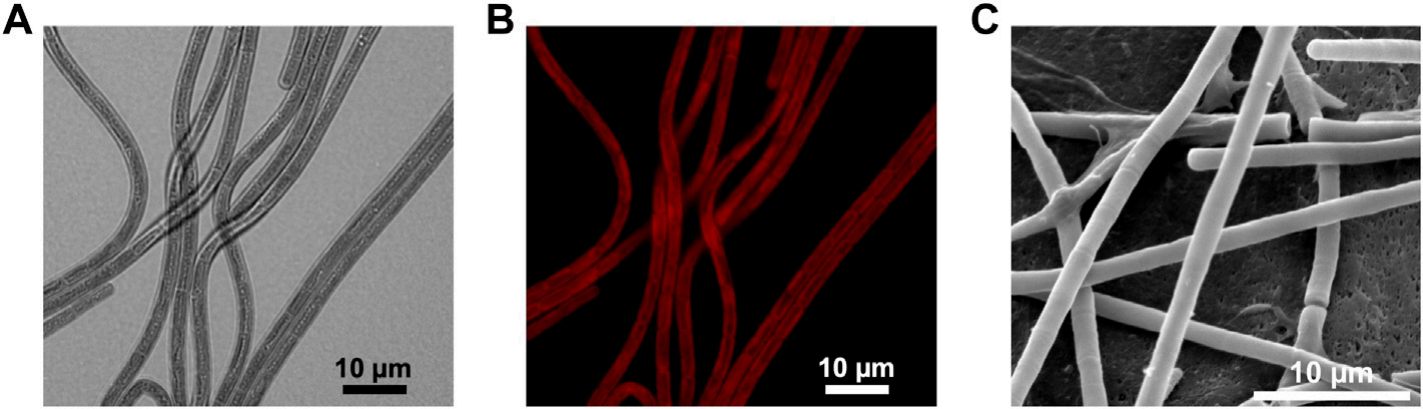
Microscopic images of *P. yuhuli* AB48. Pictures are taken with a light **(A)**, fluorescence **(B)**, scanning electron microscope (SEM) **(C)**.

*Phormidium yuhuli* AB48 grows in long, unbranched filaments and does not produce any specialized cells, such as heterocysts, under the growth conditions tested. Based on these observations, it is classified as a Section 3 cyanobacterium. Individual cells are between 2.8 and 3.5 µm wide and 5–8 µm in length, while filaments extend up to 400 µm. It was frequently observed that several parallel filaments can be located directly adjacent to one another, forming a sheet-like structure that may contribute to biofilm formation (Figures 3A,B). *P. yuhuli* AB48 cells exhibit a red autofluorescence during vegetative growth, indicating the presence of photopigments (Figure 3B). No significant gaps were observed between cells based on SEM images (Figure 3C). Extracellular polysaccharide (EPS) films can be seen surrounding some filaments in SEM images. These EPS layers likely play an important role in filament motility and tolerance to high salinity and alkalinity growth conditions.

**FIGURE 4:**
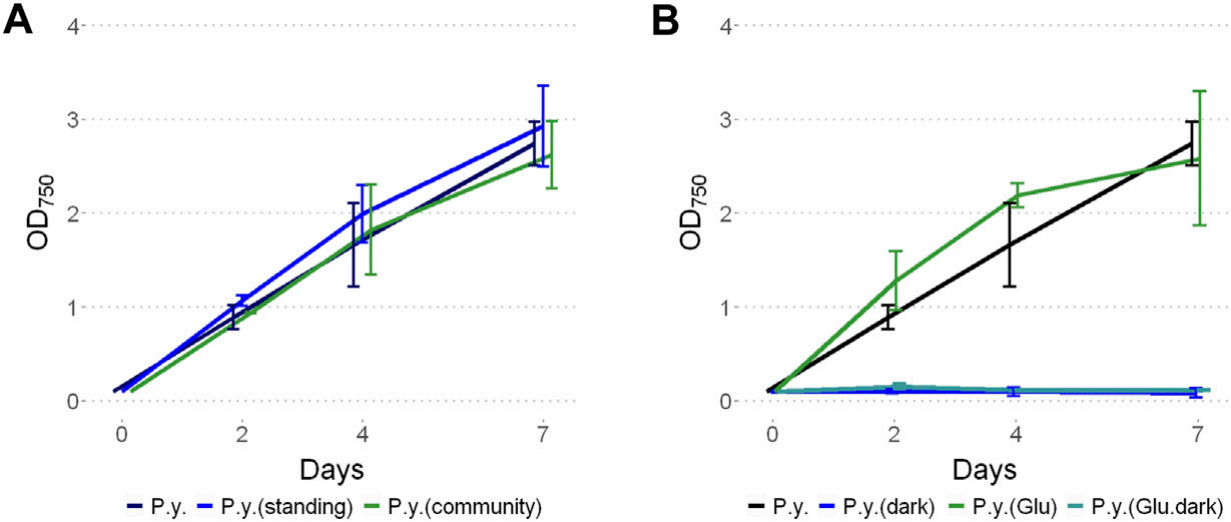
Growth of *P. yuhuli* AB48 (P.y.) under different conditions. **(A)** Comparison between shaking or standing conditions and with the mixed community. **(B)** Phototrophic growth compared to dark incubation with the addition of 10 mM glucose (“glucose”) or a combination of both. Each point represents a mean of three independent biological replicates. Error bars represent the standard error.

*Phormidium yuhuli* AB48 grew at similar rates in an axenic culture compared to a mixed community, with a doubling time of approximately 20 h (Figure 4A). Interestingly, based on a qualitative assessment, axenic cultures appear to produce noticeably more bubbles at the surface of a standing culture, in comparison to mixed community cultures. This could result from the accumulation of EPS when not consumed by co-occurring heterotrophic microorganisms. Axenic cultures were not stable over long periods of incubation and tended to crash after 7–10 days, if not passaged. In contrast, enrichment communities could last for up to 4 weeks before passaging. No difference in the growth rate was observed between standing and shaking cultures. When examined under the microscope, filaments displayed significant gliding motility, moving parallel to the direction of the filament. In filamentous cyanobacteria, motility is driven by type IV pili systems and requires the production and excretion of extracellular polysaccharides (Khayatan et al., 2015; Wilde and Mullineaux, 2015; Schuergers et al., 2017). This process occurs through extension and subsequent retraction of pilin protein fibers that drag filaments through a polysaccharide sheath. *P. yuhuli* AB48 also displayed phototactic behavior gliding in the direction of a light source (Supplementary Figure S3). When *P. yuhuli* AB48 was cultivated with additional carbon sources, such as 10 mM glucose, a slight increase in the growth rate was observed during the first days in culture (Figure 4B). This could indicate the ability to grow mixotrophically. However, when the strain was cultivated in the dark, no growth was observed, with or without glucose amendment. Similar experiments with acetate or fructose amendment showed no growth (data not shown).

To test whether *P. yuhuli* AB48 can grow diazotrophically under aerobic cultivation conditions, 100 μL cell suspension was spread on agar plates with ZM or on agar plates with nitrogen-free ZM. To account for potential diurnal impacts, plates were incubated under twelve-hour light–dark cycles. After three days, the culture on ZM plates grew, while plates without a nitrogen source turned yellow, and did not show any increase in biomass, which is consistent with a chlorotic state (Supplementary Figure S2). Additional features relevant to industrial growth of *P. yuhuli* AB48 were tested including clumping, impact of dilution on cell growth, and alternative nitrogen source utilization. To test clumping behavior, a culture was transferred to a glass vial and mixed *via* a homogenizer. The cells were then observed over a 20 min period while they aggregated at the top of the vial, forming dense clumps (Supplementary Figure S4). To test whether *P. yuhuli* AB48 can also grow at lower cell concentrations, a dilution series between OD_750_ 0.1 and 0.0001 was prepared. Even at the most diluted, *P. yuhuli* AB48 was able to grow. To test growth on an alternative nitrogen source, cultures were grown for 7 days in ZM with and without nitrogen (Supplementary Figure S5). In parallel, artificial urine (AU) was added to nitrogen-free ZM. *P. yuhuli* AB48 was able grow in up to 10% AU indicating the potential to use urea from diluted urine as an alternative nitrogen source.

**FIGURE 5:**
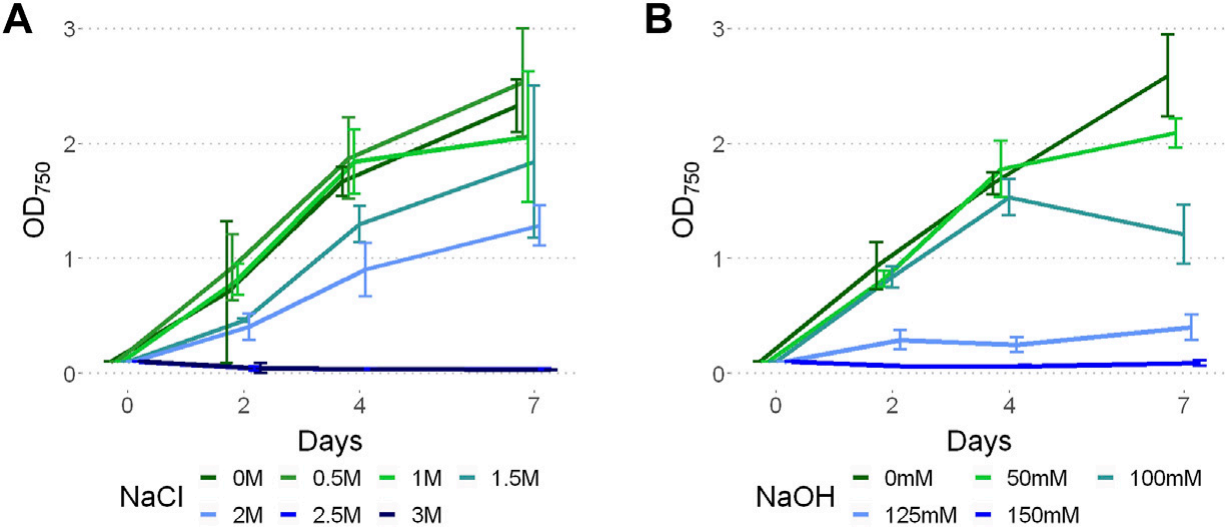
Growth of *P. yuhuli* AB48 at different concentrations of NaCl **(A)** and NaOH **(B)**. Each point represents a mean of three independent biological replicates. Error bars represent the standard error.

Limits to *P. yuhuli* AB48 growth were investigated in a series of stress tests focused on salinity, alkalinity, and antibiotic treatment. For salinity testing, the strain was grown for one week under different NaCl concentrations, ranging from 0 to 3 M (Figure 5A). The results indicate that *P. yuhuli* can grow well until 1 M NaCl. Above this concentration, a decrease in growth rate was observed. When cultivated above 2 M, no growth was observed. For alkalinity testing, cultures were grown for one week under different NaOH concentrations up to 150 mM. (Figure 5B). Interestingly, some growth inhibition was observed on day 7 at 50 and 100 mM NaOH despite unimpacted growth on days prior. When cultivated at concentrations above 125 mM NaOH, little to no growth was observed.

**FIGURE 6:**
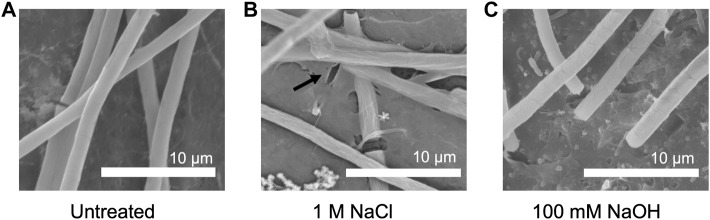
SEM pictures of *P. yuhuli* AB48 incubated in ZM **(A)**, 1 M NaCl **(B)**, or 100 mM NaOH **(C)**. Extracellular polysaccharide (EPS) films are indicated by the black arrow.

To investigate how growth under elevated NaCl and NaOH concentrations impacted cell morphology, the samples were treated for 30 min with 1 M NaCl and 100 mM NaOH, respectively, and visualized *via* SEM. In comparison to untreated cells (Figure 6A), the surface of cells treated with NaCl was noticeably less smooth (Figure 6B). Instead, small folds were visible on the filaments, potentially resulting from a change in osmotic pressure. There also appeared to be an increase in EPS production (Figure 6B), indicating a possible protection mechanism, consistent with previous observations in *Phormidium* (Gerasimenko et al., 2003). When cells were treated with NaOH (Figure 6C), cell surfaces appeared to form scales or fractures along the length of the filament.

**FIGURE 7:**
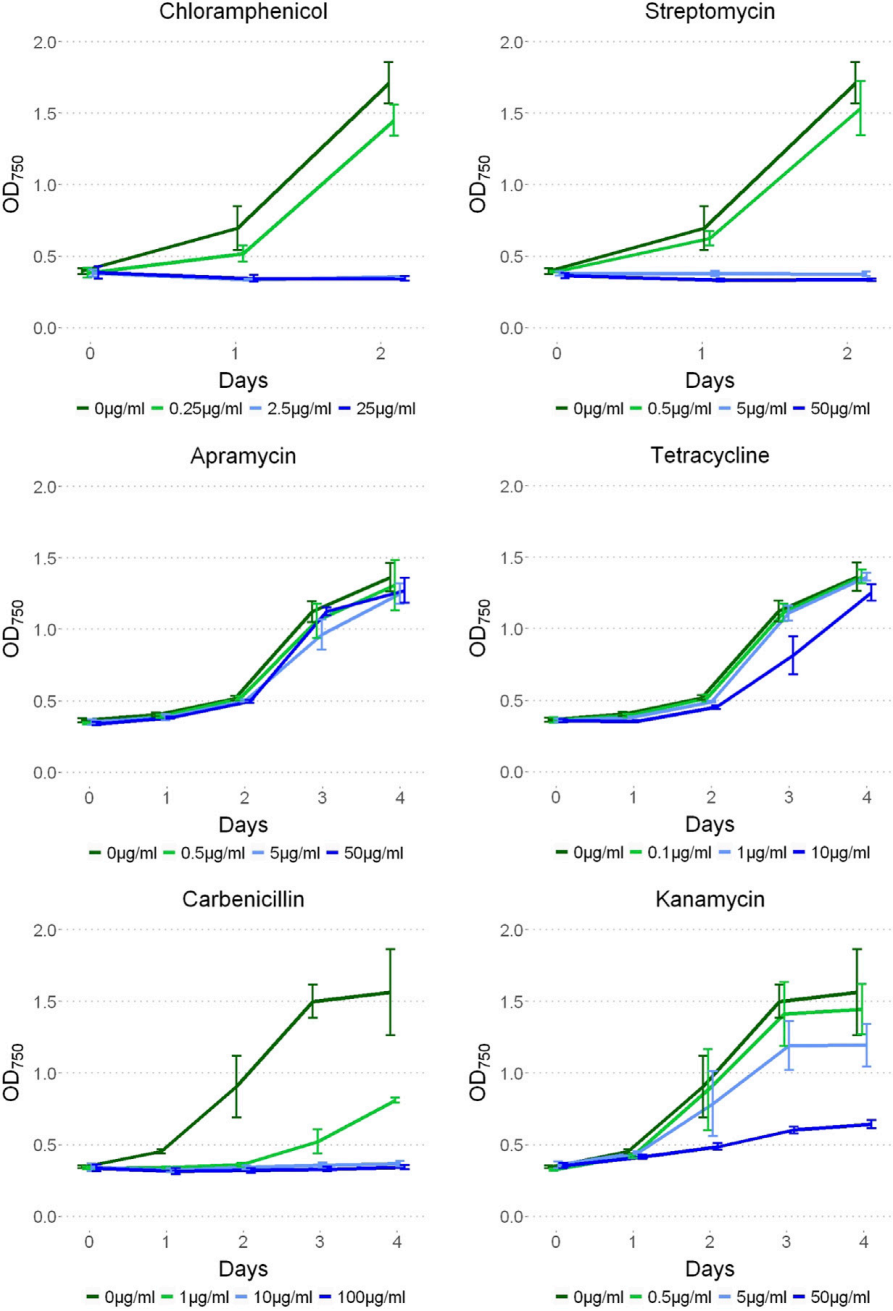
*P. yuhuli* AB48 grown on 0, 1, 10, and 100% working concentrations of streptomycin (50 μg/ml), apramycin (50 μg/ml), chloramphenicol (25 μg/ml), tetracycline (10 μg/ml), carbenicillin (100 μg/ml), and kanamycin (50 μg/ml) antibiotics. Error bars represent the standard error.

To test antibiotic sensitivity, *P. yuhuli* AB48 was cultivated under a range of concentrations of commonly used antibiotics including chloramphenicol, streptomycin, apramycin, tetracycline, carbenicillin, and kanamycin (Figure 7). Optical density readings were comparable between the no antibiotic control and apramycin at all concentrations suggesting that *P. yuhuli* shows strong resistance. Likewise, tetracycline proved ineffective at inhibiting growth at all concentrations other than 100% working concentration where significant growth was still observed. Streptomycin and chloramphenicol effectively prevented growth at concentrations 10% and higher, with carbenicillin showing a similar pattern of inhibition. Kanamycin did not prevent growth at 100% working concentrations. This resistance was also observed with gentamicin, which is also an aminoglycoside (data not shown). These phenotypes are noteworthy, as kanamycin was used as a selection marker in the engineering of *P. lacuna*, indicating that this resistance mechanism may not be shared by all *Phormidium* species.

To investigate the chemical composition and protein content of *P. yuhuli* AB48 biomass, several liters of culture were harvested, washed and sent to a chemical analysis laboratory (MB Labs, Sydney, Canada). The results indicate an N:P:K (nitrogen: phosphorus: potassium) ratio of approximately 7:4:3 and a protein content of over 40% (Table 2). Importantly, genome annotations and mass spectrometry confirmed that *P. yuhuli* AB48 does not produce any of a panel of 13 cyanobacterial toxins, including seven microcystins, anatoxin, nodularin, cylindrospermopsin, saxitoxin, neosaxitoxin, and domoic acid, reinforcing its potential as a food source or fertilizer component.

**TABLE 2:** Major elemental composition and protein content of *P. yuhuli* AB48. Only elements present at >0.1% are shown.

Component	Percentage (%)
Nitrogen	7.3
Sodium	4.0
Phosphorus	2.9
Potassium	1.4
Calcium	0.7
Magnesium	0.6
Iron	0.3
Aluminum	0.2
Zinc	0.1
Total protein	41.4

### Multi-omic analysis of *P. yuhuli* AB48

The closed reference genome of *P. yuhuli* AB48 was annotated to identify traits associated with carbon processing, growth under extreme conditions, nitrogen fixation potential, and other biotechnologically relevant phenotypes. The identification of genes encoding several of these traits was validated using a combination of proteomics and metabolomics.

**FIGURE 8:**
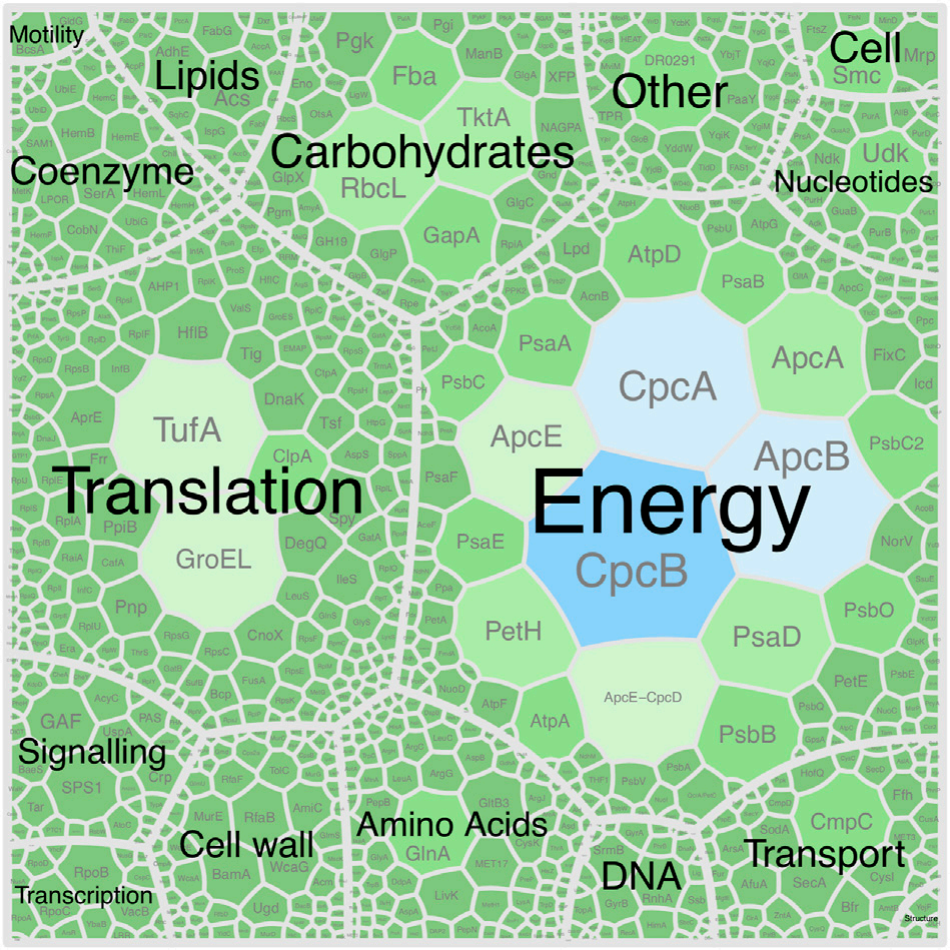
Voronoi diagram represent the *P. yuhuli* AB48 proteome. Size and color of regions indicate abundance of associated proteins determined by the proportion of assigned spectral counts. Proteins are split into functional categories defined by the COG database. The largest CpcAB and ApcBE regions represent subunits of phycocyanin and allophycocyanin.

As expected for a cyanobacterium, the genome encodes metabolic pathways and enzyme complexes required for oxygenic photosynthesis. All components of photosystem I (PSI) were identified and expressed with the exception of *PsaX*, which is unique to thermophilic cyanobacteria (Vanselow et al., 2009). All components of photosystem II (PSII), the Calvin cycle and carboxysome assembly were also identified and expressed (Figure 8). Cyanobacterial photopigments have various biotechnological, commercial, and health properties, making them important components of an industrial chassis organism (Mandal et al., 2020). *P. yuhuli* AB48 encodes and expresses both subunits of allophycocyanin (*apcA* and *apcB*), phycocyanin (*cpcA* and *cpcB*), and phycoerythrin (*cpeA* and *cpeB*). According to spectral counts, these proteins, specifically the α- and β-subunits of phycocyanin, make up the most abundant proteins in the cell (Figure 8). In addition, we verified the biosynthesis of both β-carotene and zeaxanthin, through GC–MS, and identified several genes associated with carotenoid biosynthesis including phytoene synthase (*crtB*), phytoene desaturase (*crtI*) and zeta-carotene–forming phytoene desaturase (*carA2*). The expression of these proteins was also confirmed through proteomics.

Next, traits supporting growth under extreme conditions were investigated with an emphasis on salinity and alkalinity. There are several recognized mechanisms of salt tolerance in cyanobacteria including active and passive transport of sodium, potassium, and chloride ions in and out of the cell, accumulation of compatible solutes, increased EPS production, and variations in the proportion of saturated lipids in the cell membrane (Hagemann, 2011; Kirsch et al., 2019). The *P. yuhuli* AB48 genome encodes 21 genes related to various sodium transporters. In response to a high salinity environment, an increase in the expression of the Na^+^/H^+^ antiporter KefB (1.4-fold) was observed with a concomitant decrease in the expression of BicA, CmpC, and CmpB (1.6-fold–1.8-fold), involved in the bicarbonate transport (Supplementary Figure S6). In addition to genes related to inorganic ion transportation, variation in the expression of several genes related to metabolism and transport of compatible solutes was observed, as well as the accumulation of compatible solutes in *P. yuhuli* AB48 biomass (Hagemann, 2011; Pade and Hagemann, 2014; Kirsch et al., 2019; Klähn et al., 2021). These included a 3.4-fold increase in glycerol-3-phophate dehydrogenase, GlpA, expression and a 3.9-fold and 2.9-fold decrease in PulA and AmyA expression, which are involved in polysaccharide biosynthesis and degradation, respectively. Consistent with these observations, *P. yuhuli* AB48 accumulated glycerol (9.3-fold), d-fructose-6-phosphate (4.8-fold) and d-glucose-6-phosphate (3.1-fold), and depleted sucrose (4.2-fold) and l-glutamine (7.9-fold) (Supplementary Figure S6). Although these compounds are known to play a role in the regulation of osmotic pressure in cyanobacteria, sucrose has more commonly been observed to increase in concentration in response to high salinity (Kirsch et al., 2019). These results indicate that glycerol, fructose, and sucrose may act as compatible solutes in *P. yuhuli* AB48 and suggest that various di-saccharides and mono-saccharides can play analogous roles in salt-tolerance.

The cyanobacterial response to alkaline environments is thought to be mediated by several mechanisms, including an increased expression of transporters regulating the influx of H^+^ and HCO_3_^−^ ions, and changes to energy metabolism, as a result of the impact on the electron transport chain and the availability of inorganic carbon (Summerfield Tina and Sherman Louis, 2008). An increase in the expression of PsfB (3.5-fold), the cytochrome b559 subunit beta associated with the photosystem II reaction center was observed, as well as an increase in chromophore lyase Cpc3 (3.0-fold), involved in the assembly of phycobiliprotein complexes. The expression of several transporters was also impacted by increased salinity, including a decrease in CmpB (1.5-fold), CmpC (1.9-fold), and CmpD (1.6-fold) expression. These observations were supported by metabolomics data, which showed an increase in the cellular concentration of CO_3_^−^ (2.5-fold) and xanthopterin (4.8-fold), a yellow pigment that may be involved in protection against high-energy light (Feirer and Fuqua, 2017).

**FIGURE 9:**
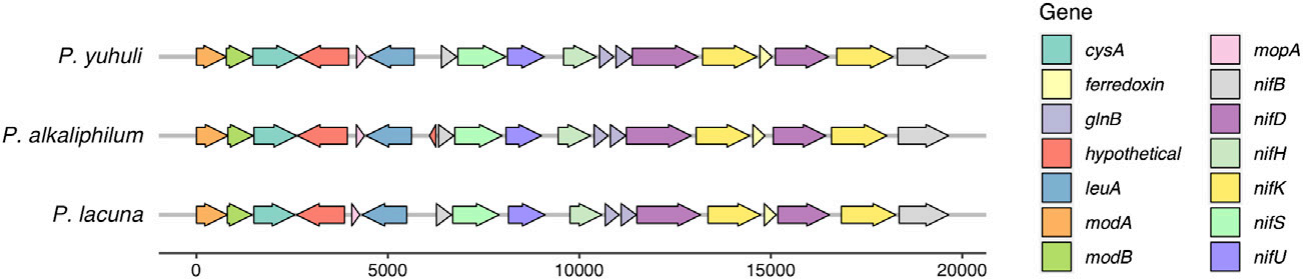
Molybdenum nitrogenase operon from *P. yuhuli* AB48 is syntenic with those of nitrogen-fixing *P. lacuna* and *P. alkaliphilum*.

Next, the genomic basis behind nitrogen-utilization phenotypes in *P. yuhuli* AB48 was investigated. Although the isolate did not grow in the nitrogen-free medium under aerobic conditions with either continuous lighting or a 12-h day–night rhythm, several *nif* molybdenum nitrogenase genes were identified in the genome and expressed in the proteome, including *nifB*, *nifD*, *nifH*, *nifK*, *nifS*, and *nifU* (Esteves-Ferreira et al., 2017). These genes are arranged in an operon sharing synteny with *Phormidium alkaliphilum* and *Phormidium lacuna,* both of which appear to fix nitrogen (Nies et al., 2017; Ataeian et al., 2021) (Figure 9). In non-heterocystous nitrogen-fixing cyanobacteria, it is thought that either special or temporal separation of photosynthesis and nitrogen fixation, and the creation of low-oxygen area in a culture, protects oxygen-sensitive nitrogenases (Bergman et al., 1997). Spatial separation and localized oxygen depletion may be enabled by the formation of dense biofilms, a condition not tested here. It remains to be determined under what growth conditions *P. yuhuli* AB48 is capable of nitrogen fixation. Consistent with the observed capacity to grow on urine as a nitrogen source, a complete urease operon and transport system encoded by *ureA-G urtA-E*, respectively, were identified and expressed*.* Phosphorus is also an important component of synthetic and organic fertilizers (Coppens et al., 2016). *P. yuhuli* AB48 expresses multiple copies of a polyphosphate kinase (PPK) (Sanz-Luque et al., 2020), indicating that it may also provide a mechanism of accumulating and storing phosphate for application as a fertilizer.

The genome encodes and expresses the pilins *pilT1* and *pilT2*, both known to be essential for gliding motility in filamentous cyanobacteria (Khayatan, Meeks and Risser, 2015; Wilde and Mullineaux, 2015), as well as several copies of genes involved in signaling related to chemotaxis and phototaxis, including chemotaxis genes *cheY*, *cheA*, *cheW*, and several methyl-accepting chemotaxis proteins (MCP) (Jakob et al., 2019). Finally, the observed resistance to tetracycline and apramycin was supported by the identification of genes encoding the tetracycline resistance protein, TetA, as well as multiple copies of the *acrAB* multidrug transporter known to be involved in aminoglycoside resistance (Garneau-Tsodikova and Labby, 2016).

## Discussion

In this study, we described the initial morphological, physiological, and genomic characterization of the filamentous cyanobacterium *Phormidium yuhuli* AB48. Similar to other members of the *Phormidium* genus, *P. yuhuli* AB48 harbors distinguishing growth characteristics, positioning as a suitable strain for industrial applications. When cultivated under standard laboratory conditions, its doubling time is around 20 h, which is comparable to well-studied cyanobacteria such as *Synechocystis* sp. PCC 6803 when grown under similar conditions (Koch et al., 2020). In contrast to *Synechocystis* sp. PCC 6803, *P. yuhuli* AB48 thrives under high salinity and alkaline conditions (Zavřel et al., 2017). The analysis of its genome, proteome, and metabolome identified various mechanisms by which *P. yuhuli* AB48 tolerates these harsh conditions, mechanisms that are consistent with other members of the *Phormidium* genus inhabiting soda lakes and other high-salinity environments. Leveraging these features could prove valuable not only in the minimization of contamination in large-scale cultivation, but also in the deliberate regulation of metabolic activity and output.

Although *P. yuhuli* AB48 does not fix nitrogen under the aerobic cultivation conditions tested, it does encode and express molybdenum nitrogenase subunits. It has been observed that either temporal or spatial separation of oxygenic photosynthesis and nitrogen fixation is required to fix nitrogen in non-heterocystous cyanobacteria and dense biofilms may promote the process along an oxygen gradient (Bergman et al., 1997; Bothe et al., 2010; Mishra et al., 2019). Interestingly, both *Phormidium lacuna* and Candidatus *Phormidium alkaliphilum* have been reported to fix nitrogen (Nies et al., 2017; Ataeian et al., 2021). Cyanobacterial nitrogenases have also been shown to be involved in hydrogen production in aerobic environments (Bothe et al., 2010; Mishra et al., 2019). Whether this mechanism is active in *P. yuhuli* AB48 remains to be determined. Understanding the role of *nif* expression in *P. yuhuli* AB48 remains important unknown relevant to its development as a potential fertilizer supplement.

*Phormidium yuhuli* AB48 manifests several features that make it a promising microbial cell factory for industrial-scale bioproduction. First, the strain grows at an equal rate in co-culture and in isolation. This feature makes detailed metabolic modeling and pathway engineering more tractable (Jiao et al., 2020), while enabling the design of microbial consortia tuned to grow in co-culture *P. yuhuli* AB48. An interesting phenotype displayed by axenic *P. yuhuli* AB48 cultures, which has relevance to industrial application, is their instability over extended periods of time. This characteristic of axenic cyanobacterial cultures has been observed in several strains of *Synechococcus* and associated with the accumulation of reactive oxygen species, organic carbon, or other metabolites, which becomes toxic if not converted or consumed by heterotrophic members of the community (Cohen et al., 2014; Christie-Oleza et al., 2017; Vu et al., 2018). However, given that *P. yuhuli* AB48 can thrive under the non-sterile conditions of an industrial photobioreactor and can be maintained as the dominant member of an enrichment (∼95%), and it is already poised for scale-up production alone or in the community. *Phormidium yuhuli* AB48 also thrives as a dense biofilm or in a suspended culture, with robust growth under shaking or standing conditions. Other filamentous strains, such as *Anabaena*, are typically incubated at low shaking speed to avoid damage resulting from shearing forces (Mandakovic et al., 2016). The robustness of *P. yuhuli* AB48 allows growth under a variety of large-scale photobioreactor formats, some of which require intense stirring for thorough mixing. Furthermore, its ability to move and clump in response to environmental stimuli could be used for self-flocculation. This phenomenon has been explored as an easy and inexpensive biomass harvesting method further reducing bioproduction costs (Panuschka et al., 2019; Price et al., 2022). Indeed, because some of these characteristics are shared by a range of filamentous cyanobacterium, their industrial application is gaining traction, with recent studies indicating that biofilms can support very high conversion rates (Heuschkel et al., 2020) and dense biomass production (Guljamow et al., 2017).

Chemical composition and metabolite analysis of *P. yuhuli* AB48 revealed several promising features for bioproduction including its use as a food source or fertilizer supplement. In addition to the relatively high protein content, *P. yuhuli* AB48 produces abundant photopigments, as well as zeaxanthin, a carotenoid with promising health benefits, and accumulates both nitrogen and phosphorus. Moreover, the isolate did not encode or produce any known cyanobacterial toxins. Taken together, these results point to the potential application of *P. yuhuli* AB48 as a single-cell protein (SCP) source for direct consumption, similar to *Spirulina* (Lafarga et al., 2020). Microalgae can also be used as an organic fertilizer supplement (Coppens et al., 2016). Although *P. yuhuli* AB48 did not fix nitrogen under the current growth conditions, its capacity to use urine as a nitrogen source and potential to accumulate phosphorous indicates a potential application in wastewater conversion and nutrient recovery in agricultural settings including remote communities. The accumulation of mono-, di-, and polysaccharides also makes *P. yuhuli* AB48 a promising candidate for co-culture engineering.

In conclusion, we have described a novel cyanobacterial isolate *P. yuhuli* AB48 that forms dense biofilms under high salinity and alkalinity conditions at industrial scales. The strain encodes numerous traits indicating its potential to become a useful microbial cell factory. Future efforts to model the metabolic network of *P. yuhuli* AB48 under different growth conditions are needed to develop its capacity for bioproduction. At the same time, genetic tools are needed to establish this isolate as a chassis organism. Although the transformation of cyanobacteria has typically been challenging, as a result of extensive restriction modification systems, the closely related *P. lacuna* was successfully transformed using an integrative plasmid system, relying on endogenous recombinases. This strategy, as well as several broad-host-range vectors, may prove suitable for the engineering of *P. yuhuli* AB48. In addition, the presence of endogenous DNA surveillance and restriction modification systems, as well as a plasmid, in the *P. yuhuli* AB48 genome provide alternative paths forward in the development of genetic tools needed for pathway engineering, with the ultimate goal of sustainable bioproduction.

## Data Availability

The datasets presented in this study can be found in online repositories. The names of the repository/repositories and accession number(s) can be found in the article/Supplementary Material.
