# Corrigendum: The survivor strain: Isolation and characterization of *Phormidium yuhuli* AB48, a filamentous phototactic cyanobacterium with biotechnological potential

**DOI:** 10.3389/fbioe.2023.1160235

**Published:** 2023-03-14

**Authors:** Moritz Koch, Avery J. C. Noonan, Yilin Qiu, Kalen Dofher, Brandon Kieft, Soheyl Mottahedeh, Manisha Shastri, Steven J. Hallam

**Affiliations:** ^1^ Department of Microbiology and Immunology, University of British Columbia, Vancouver, BC, Canada; ^2^ Genome Science and Technology Program, University of British Columbia, Vancouver, BC, Canada; ^3^ ECOSCOPE Training Program, University of British Columbia, Vancouver, BC, Canada; ^4^ AlgaBloom International Ltd., Richmond, BC, Canada; ^5^ Graduate Program in Bioinformatics, University of British Columbia, Vancouver, BC, Canada; ^6^ Life Sciences Institute, University of British Columbia, Vancouver, BC, Canada

**Keywords:** cyanobacteria, phormidium, photobioreactor, isolate, stress tolerance, oscillatoriales

In the published article, there was an error. The introduction to this manuscript includes a typographical error which misstates the maximum salinity and pH previously observed in related cyanobacterial species. This error does not impact any analyses or conclusions reached in this manuscript.

A correction has been made to **Introduction**, Paragraph 2. This sentence previously stated:

“*Phormidium* are common denizens of soda lakes and other high-salinity environments, forming dense biofilms in conditions up to 3 M salinity and pH 13.5 (Kupriyanova et al., 2016; Samylina et al., 2014; Ataeian et al., 2021).”

The corrected sentence appears below:

“*Phormidium* are common denizens of soda lakes and other high-salinity environments, forming dense biofilms in conditions up to 200 g/L total salinity and pH 11.2 (Kupriyanova et al., 2016; Samylina et al., 2014; Ataeian et al., 2021).”

There was another error in the published article. The “Morphology and growth characteristics” subsection of this manuscript includes a typographical error which misstates the maximum NaOH concentration used for alkalinity testing. This error does not impact any analyses or conclusions reached in this manuscript.

A correction has been made to **Results**, **Morphology and growth characteristics**, Paragraph 4. This sentence previously stated:

“For alkalinity testing, cultures were grown for one week under different NaOH concentrations up to 200 mM (Figure 5B).”

The corrected sentence appears below:

“For alkalinity testing, cultures were grown for one week under different NaOH concentrations up to 150 mM (Figure 5B).”

And finally, there was a third error in the published article. The “Strain isolation” subsection of this manuscript includes a typographical error which misstates the maximum pH used for strain isolation.

A correction has been made to **Strain isolation**, Paragraph 1. This sentence previously stated:

“The cells growing under high salinity (672 mM NaCl) and pH 13 (100 mM NaOH), which was the harshest condition in which growth was observed, were selected and passaged several times in a fresh ZM medium.”

The corrected sentence appears below:

“The cells growing under high salinity (672 mM NaCl) and pH (100 mM NaOH), which was the harshest condition in which growth was observed, were selected and passaged several times in a fresh ZM medium.”

The authors apologize for these errors and state that this does not change the scientific conclusions of the article in any way. The original article has been updated.

