# Supplementary material for: The survivor strain: isolation and characterization of Phormidium yuhuli AB48, a filamentous phototactic cyanobacterium with biotechnological potential
Source: Front Bioeng Biotechnol. 2022 Aug 15;10:932695. doi: 10.3389/fbioe.2022.932695 (PMC9420970; doi:10.3389/fbioe.2022.932695)
Supplement: Supplementary file 2 [file DataSheet2.docx]

Supplementary Material

# Supplementary Data

Supplementary data and data processing scripts are included in an associated .zip file or have been uploaded to public databases as specified in the manuscript. The following table shows contents of compressed directory and a brief description of each file.

| **File Name** | **Description** |
| --- | --- |
| Koch_RMarkdown_Phormidium_yuhuli_AB48.Rmd | R code used to create Figures 1, 4, 5, 7, 8, 9 and S6 |
| p_yuhuli_annotation.csv | *Phormidium yuhuli* AB48 genome annotation |
| 16s_community_profiling_data.csv | Taxonomic classification of 16S rRNA amplicon sequence data (**Figure 1**) |
| tree_genome_info.tsv | NCBI and GTDB taxonomy of genome included in phylogenetic tree (**Figure 2**) |
| community_growth_curve.csv | OD_750_ data from growth experiments (**Figure 4**) |
| mixotrophic_growth_curve.csv | OD_750_ data from growth experiments (**Figure 4**) |
| NaCl_growth_curve.csv | OD_750_ data from growth experiments (**Figure 5**) |
| NaOH_growth_curve.csv | OD_750_ data from growth experiments (**Figure 5**) |
| antibiotic_resistance_data.csv | OD_750_ data from growth experiments (**Figure 7**) |
| p_yuhuli_spectral_data.csv | Proteomics data (assigned spectral counts) from wild-type *Phormidium yuhuli* AB48 (**Figure 8**) |
| nif_synteny_input.csv | Gene position of *nif* cluster genes as input for synteny plot (**Figure 9**) |
| stress_test_spectral_data.csv | Proteomics data (assigned spectral counts) from *Phormidium yuhuli* AB48 under varying growth conditions. |
| stress_test_metabolomics_data.csv | Metabolomics data (m/Z) from *Phormidium yuhuli* AB48 under varying growth conditions. |

# Supplementary Figures


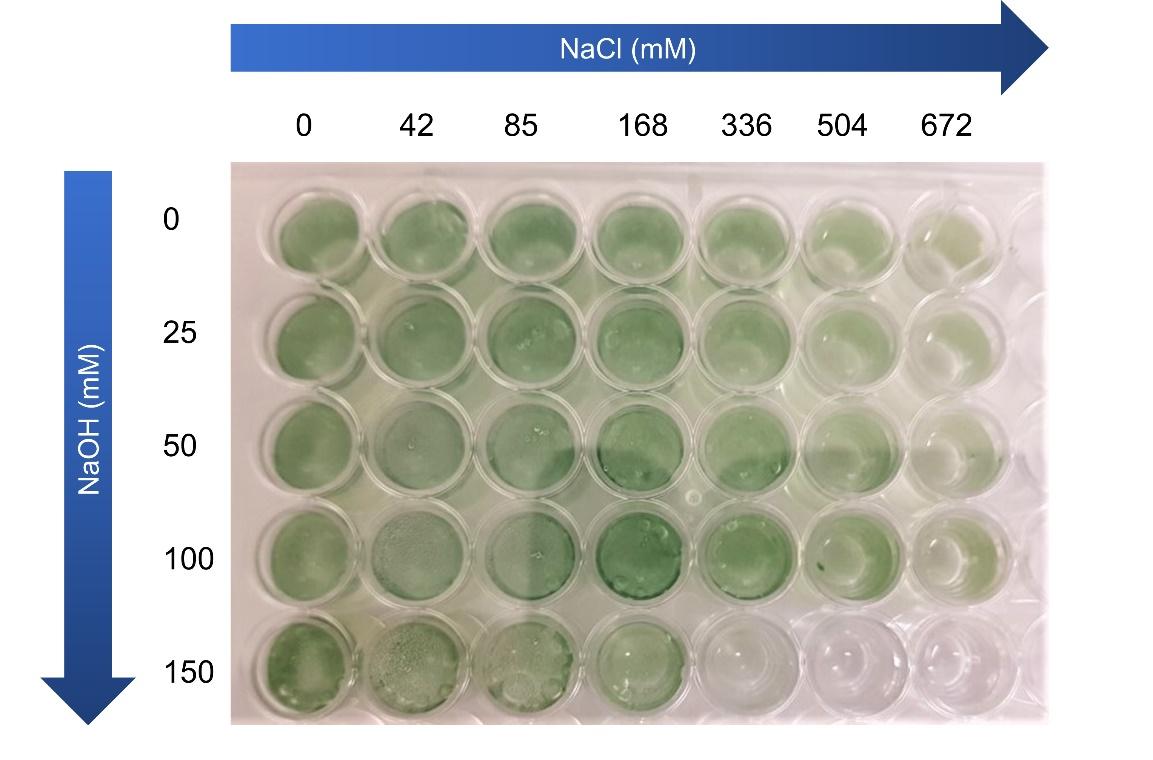


**Supplementary Figure 1.** Incubation matrix for the isolation of *P. yuhuli* AB48. Each row contained increasing concentrations of NaOH (0-150 mM), while each column contained increasing amounts of NaCl (0-672 mM).


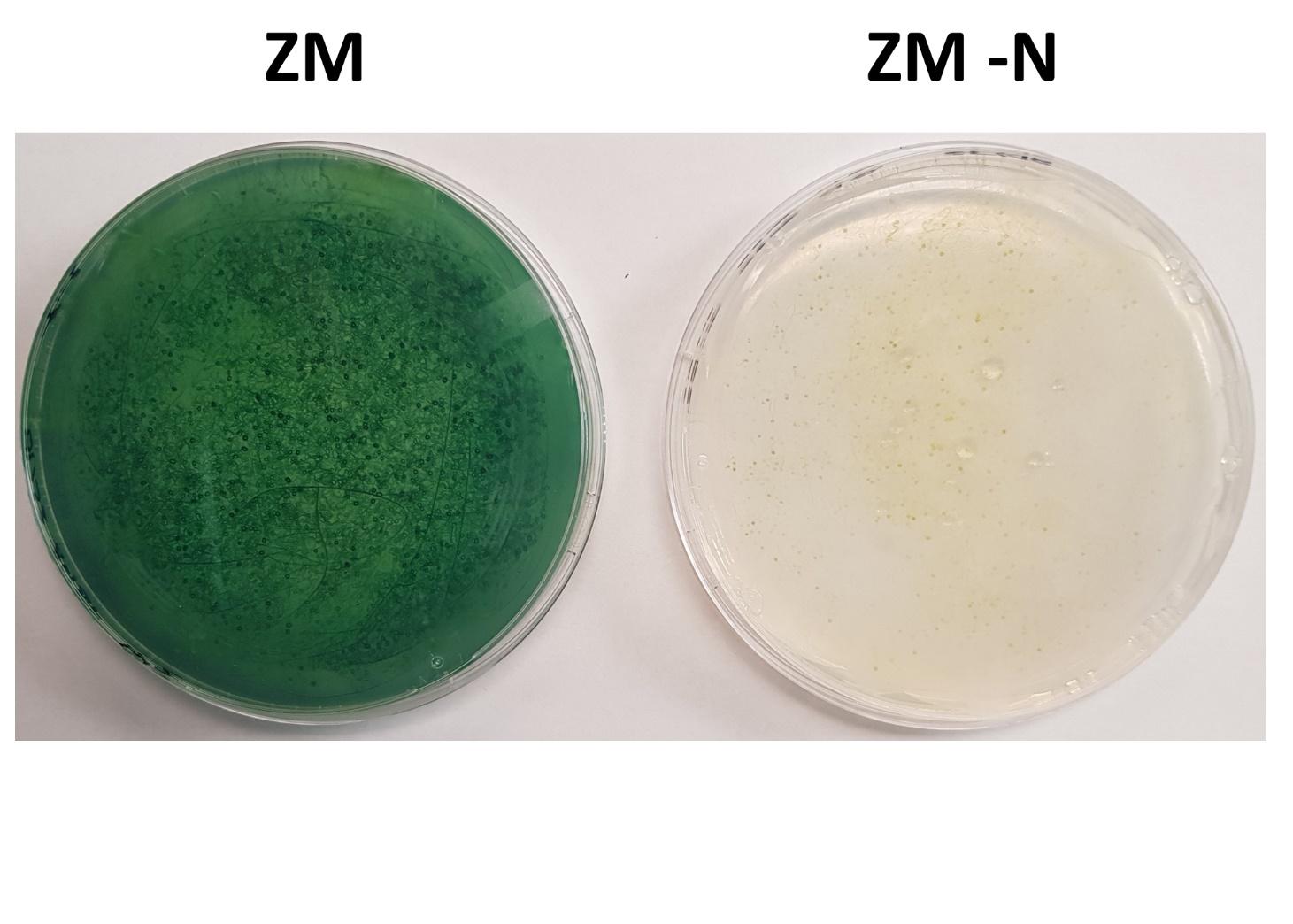


**Supplementary Figure 2.** Growth of *P. yuhuli* AB48 on agar plates with and without nitrogen. *P. yuhuli* AB48 (100 µl) was applied to agar plates of Zarrouk medium (ZM) with or without nitrogen, left and right, respectively. The plates were incubated under alternating day-night light regime.


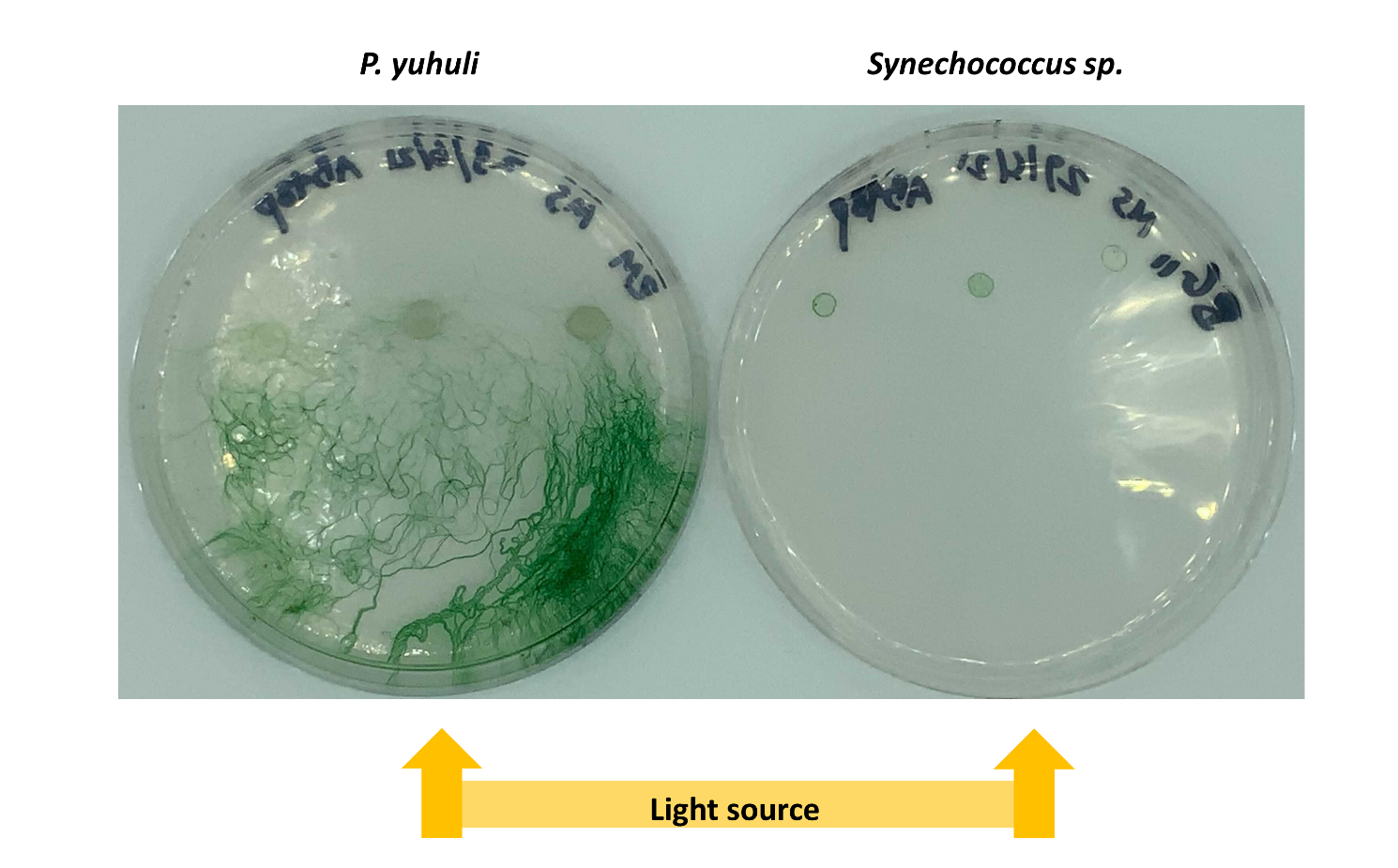


**Supplementary Figure 3.** Phototaxis of *P. yuhuli* AB48 and *Synechococcus sp.* UTEX 2973 on agar plates. The cultures were applied as three drops of 5 µl. The plates were kept in a box where light was only able to hit the agar plates from one side.


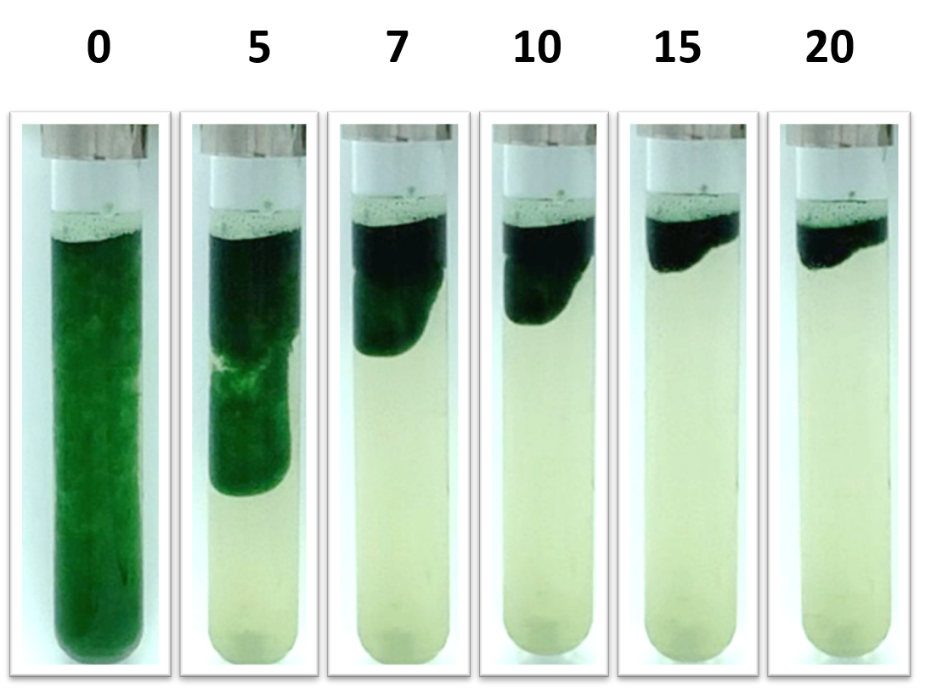


**Supplementary Figure 4.** Clumping phenotype in liquid cultures. *P. yuhuli* AB48 cultures were mixed and the vial was left standing for subsequent 20 minutes.


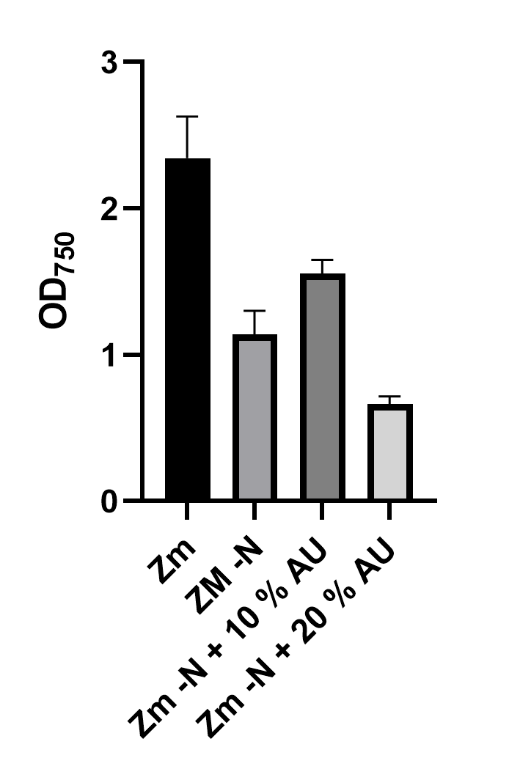


**Supplementary Figure 5.** Growth with alternate nitrogen sources. *P. yuhuli* AB48 was cultivated in Zarrouk medium with (ZM) or without (ZM -N) nitrogen. Some of the samples contained additionally artificial urine (AU). The original OD_750_ was 0.1 and samples were taken after incubation for one week. Each bar represents a mean of three independent biological replicates.

**Supplementary Figure 6.** Impact of high-salinity and alkaline conditions on *P. yuhuli* AB48 proteomics and metabolomics. Boxplots show relative of selected proteins and metabolites, based on peptide counts and m/Z, respectively. Boxes contain values from 4 samples.
